# 26 Formalizing Burn Nurse Education, a Literature Review on Nursing Fellowships and Discussion for Furthering Training

**DOI:** 10.1093/jbcr/irae036.026

**Published:** 2024-04-17

**Authors:** Alison Hao-Smith, Elisabeth Fletcher, Daud Lodin

**Affiliations:** New York-Presbyterian Weill Cornell Medical Center, New York, New York; University of Wisconsin Hospitals and Clinics, Madison, Wisconsin; Florida Atlantic University College of Medicine, West Palm Beach, Florida; New York-Presbyterian Weill Cornell Medical Center, New York, New York; University of Wisconsin Hospitals and Clinics, Madison, Wisconsin; Florida Atlantic University College of Medicine, West Palm Beach, Florida; New York-Presbyterian Weill Cornell Medical Center, New York, New York; University of Wisconsin Hospitals and Clinics, Madison, Wisconsin; Florida Atlantic University College of Medicine, West Palm Beach, Florida

## Abstract

**Introduction:**

With the recent implementation of the exam for Certified Burn Registered Nurses (CBRN) by the Board of Certification for Emergency Nursing (BCEN), a curriculum for burn nursing has been developed to better strengthen and standardize burn nursing care. Given the depth of the curriculum and the overall demand of becoming a burn nurse, the need for creating an educational track for burn nursing becomes apparent. While most burn centers utilize a preceptorship model to help train registered nurses to become burn nurses, a nationally recognized fellowship has not been developed. This study discusses the outcome of formalized nursing fellowships and how it could impact burn nursing training.

**Methods:**

This is a literature review of published data from 2000 to 2023. This review utilizes PubMed to search indexed articles, using the terms “nursing fellowship”, “nurses fellowship”, and “nurse fellowship”. The articles were examined for content and outcomes. Articles discussing fellowships for advanced practitioners and those that did not discuss the direct benefit of their programs were excluded. The results were collated based on specialty, type of fellowship, and both the positive and negative outcomes discussed in these articles.

**Results:**

Eleven articles were identified related to nursing fellowships. These involved formalized training programs in primary care, addiction medicine, leadership, research, gastroenterology, perioperative care, and critical care. Many articles discussed the benefit of increased knowledge and student performance in fellowship graduates. From an expense perspective, the articles identified cost benefits from employee retention and prevention of error resulting in decreased cost of hospitalization and litigation. One publication discussed an improvement in measured professionalism inventory scores in graduates. Several articles commented on nursing retention, which noted that in some instances, units were able to improve retention by over 40 percent.

**Conclusions:**

The development of burn nursing certification paves the way for the development of a burn nursing fellowship. This literature review identifies benefits that other specialties have had with the development of formalized nursing fellowships, which include increased knowledge, improved skills, fewer errors, and increased retention.

**Applicability of Research to Practice:**

This study discusses the potential to improve burn nursing education in a formalized setting, intended to improve nursing knowledge and skills, and ultimately improve outcomes.

